# Relationship between physical activity and chronic obstructive pulmonary disease: a cross-sectional study

**DOI:** 10.3389/fpubh.2025.1583265

**Published:** 2025-05-16

**Authors:** Ziwei Kou, Yaoyao Wang, Wanming Hao, Yanmiao Li, Xinjuan Yu, Yinan Li, Yulu Zhong, Entong Gong, Tao Wang, Wei Han

**Affiliations:** ^1^Qingdao Medical College, Qingdao University, Qingdao, China; ^2^Department of Respiratory and Critical Medicine, Qingdao Municipal Hospital, University of Health and Rehabilitation Sciences, Qingdao, China; ^3^Clinical Research Center, Qingdao Key Laboratory of Common Diseases, Qingdao Municipal Hospital, University of Health and Rehabilitation Sciences, Qingdao, China; ^4^Graduate School, Dalian Medical University, Dalian, Liaoning, China; ^5^School of Health and Life Sciences, University of Health and Rehabilitation Sciences, Qingdao, China; ^6^Department of Respiratory and Critical Medicine, Qingdao Key Laboratory of Common Diseases, Qingdao Municipal Hospital, University of Health and Rehabilitation Sciences, Qingdao, China

**Keywords:** chronic obstructive pulmonary disease, physical activity level, physical activity pattern, retrospective study, NHANES

## Abstract

**Background:**

This study explores the association between physical activity (PA) levels and patterns during adulthood and chronic obstructive pulmonary disease (COPD).

**Methods:**

Data from the National Health and Nutrition Examination Survey (NHANES) 2007–2018 was analyzed. A total of 34,392 participants were included. Three physical activity levels groups were categorized: insufficiently active (individuals not meeting the criteria for “Sufficiently active” or “HEPA active”), sufficiently active ((≥3 days of vigorous activities (≥480 MET-min/week), or ≥5 days of moderate activities /walking (≥600 MET-min/week), or ≥5 days of combined activities (≥600 MET-min/week)), HEPA active ((≥3 days of vigorous activities (≥1,500 MET-minutes/week), or ≥7 days of combined activities (≥3,000 MET-min/week)). Five PA patterns groups were categorized: vigorous work activity, moderate work activity, walk/bicycle for transportation, vigorous recreational activity, moderate recreational activity. The relationship between PA and COPD was explored using a multivariable logistic regression model, restricted cubic spline (RCS) analysis, and stratified analysis.

**Results:**

Compared to insufficiently active individuals, being sufficiently active (OR: 0.86, 95% CI = 0.75–0.98, *p* = 0.025) and HEPA active (OR: 0.84, 95% CI = 0.73–0.96, *p* = 0.010) were associated with lower COPD prevalence. Compared to those lacking corresponding PA patterns, low-level (OR: 1.35, 95% CI = 1.12–1.62, *p* = 0.002) and sufficient (OR: 1.19, 95% CI = 1.05–1.35, *p* = 0.006) moderate work activities (OPA) were linked to higher COPD prevalence. Sufficient transportation-related physical activities (TPA) (OR: 0.72, 95% CI = 0.59–0.89, *p* = 0.003), sufficient vigorous recreational activities (RPA) (OR: 0.68, 95% CI = 0.55–0.85, *p* < 0.001), low-level moderate RPA (OR: 0.77, 95% CI = 0.66–0.90, *p* = 0.001), and sufficient moderate RPA (OR: 0.71, 95% CI = 0.61–0.84, *p* < 0.001) were all significantly associated with lower COPD prevalence.

**Conclusion:**

In adulthood, TPA and RPA were associated with a lower COPD prevalence, while OPA were associated with a higher COPD prevalence. However, COPD patients might become less active because of their symptoms, which may influence study results. Increasing TPA/RPA proportion in total PA could be a potential COPD prevention strategy, but causal evidence requires further validation.

## 1 Introduction

Chronic obstructive pulmonary disease (COPD) is a prevalent chronic disease that can be prevented and managed, characterized by persistent respiratory symptoms and airflow limitation (1). According to the data from the World Health Organization (WHO), COPD is currently the third leading cause of death worldwide (2). With the increasing smoking rates in developing countries and the aging population in high-income countries, it is estimated that by 2060, over 5.4 million people will die annually from COPD and its related diseases. Therefore, COPD has emerged as a pressing global health concern that demands immediate attention.

Numerous studies had shown that physical activity (PA) could significantly reduce the risk of disease exacerbation, hospitalization, and mortality in patients with COPD, making it the preferred non-pharmacological intervention for COPD (3, 4). According to the WHO, it was highlighted that one-third of adults worldwide lacked sufficient PA, which ranked among the top 10 leading risk factors for global mortality (5). And recent studies indicated that PA was associated with an increased risk of premature death and the development of various chronic diseases, including respiratory diseases, cardiovascular diseases, diabetes, and cancer (6, 7). The study conducted by Garcia et al. (8) revealed a dose-response relationship between low levels of PA and accelerated decline in lung function, compared to high levels of PA. Another population-based study found that maintaining regular PA could prevent a decline in lung function and reduce the risk of COPD occurrence (9). Therefore, the changes in PA habits may potentially be associated with alterations in cardiovascular and respiratory health.

PA refers to any body movement caused by the contraction of skeletal muscles that results in energy expenditure. It includes a range of activities in various domains such as recreational physical activities (RPA), occupational-related physical activities (OPA) or transportation-related physical activities (TPA). PA can help maintain and improve cardiovascular health, reduce the risk of obesity and related complications, and prolong lifespan (2). And RPA includes a range of recreational activities, including fitness exercises, brisk walking, swimming, volleyball, free running, and basketball. OPA is defined as paid or unpaid work, household chores, or yard work. TPA is defined as walking or cycling to and from a certain place. Previous studies had already demonstrated a positive correlation between PA and lung capacity in individuals (10, 11). However, the existing researches had primarily focused on the impact of PA as a lifestyle choice or solely on specific COPD populations, while researches on the association between PA levels and patterns with COPD were limited. Therefore, this study conducted a cross-sectional analysis based on NHANES 2007–2018 data to explore the relationships between different PA levels and patterns with COPD.

## 2 Methods

### 2.1 Study design and population

The National Health and Nutrition Examination Survey (NHANES) is a series of cross-sectional surveys conducted by the National Center for Health Statistics (NCHS), which is part of the Centers for Disease Control and Prevention (CDC) in the United States. These cross-sectional surveys were conducted every 2 years, using a stratified multistage probability sampling method to obtain a representative sample of the non-institutionalized population in the United States for each survey cycle. The investigation involved interviews and physical examinations, which were conducted by trained medical personnel who were responsible for carrying out inspections and laboratory tests. And the NHANES survey protocol was approved by the Research Ethics Review Committee of the NCHS in the United States and obtained informed consent from all participants.

Our study utilized the NHANES data from 2007 to 2018, spanning six consecutive cycles. And this study was conducted on adults aged ≥20 years, excluding pregnant women, participants with incomplete PA questionnaires, and those lacking information on COPD diagnosis.

**Figure 1:**
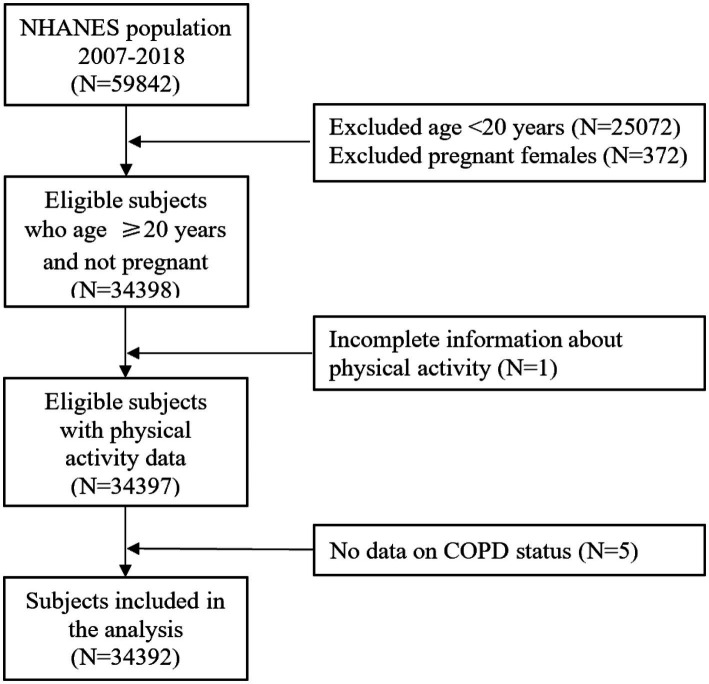
A flow chart of inclusion and exclusion of study participants.

COPD was defined as a combination of three self-reported COPD outcomes, including emphysema, chronic bronchitis, and COPD. If participants answered “yes” to the following question in the standardized medical condition questionnaire administered during individual interviews, they would be classified as having COPD: “Have you received a diagnosis of COPD/emphysema/chronic bronchitis?” Ultimately, a total of 34,392 participants were included for this retrospective study analysis (Figure 1).

### 2.2 PA

The PA status of participants in the study was assessed using the Global Physical Activity Questionnaire (GPAQ). And the questionnaire provided interview data on participants’ vigorous work activities, moderate work activities, TPA (walking and cycling), vigorous recreational activities, and moderate recreational activities, as well as reporting the frequency of PA within a week and the duration of each PA session (12). The intensity of exercise is usually expressed in terms of metabolic equivalent (MET), which refers to the ratio between the energy expended during PA and the energy expended at rest (13, 14). The MET values vary depending on the type of exercise, and NHANES provides recommended MET values for each type of PA. Moderate-intensity work or recreational activities are defined as having a MET value of 4, while high-intensity work or recreational activities are defined as having a MET value of 8. TPA is defined as having a MET value of 4 (15, 16). And we quantified PA of each participant by calculating their weekly MET-minutes (MET-min/week) based on the reported frequency and duration of PA within a week. The calculation formula was as follows: PA (MET-min/week) = frequency per week of each PA × duration × MET. The total amount of PA was defined as the sum of OPA, TPA and RPA (17).

According to the 2020 “Guidelines on Physical Activity and Sedentary Behavior” by the WHO, individuals who engaged in moderate-intensity aerobic PA for a minimum of 150 min per week, or vigorous-intensity aerobic PA for at least 75 min per week, or an equivalent combination of moderate and vigorous intensity activities totaling at least 150 min per week were considered as meeting the guidelines (18–20). Considering the total PA level, we classified participants into the following categories: (1) Insufficiently active: Those individuals who not meet criteria for Categories 2 or 3. (2) Sufficiently active: 3 or more days of vigorous activity of at least 20 min per day OR 5 or more days of moderate-intensity activity or walking of at least 30 min per day OR 5 or more days of any combination of walking, moderate-intensity or vigorous intensity activities achieving a minimum of at least 600 MET-min/week. (3) HEPA (Health Enhancing Physical Activity) active: vigorous-intensity activity on at least 3 days achieving a minimum of at least 1,500 MET-minutes/week OR 7 or more days of any combination of walking, moderate-intensity or vigorous intensity activities achieving a minimum of at least 3,000 MET-minutes/week (17, 21). To further explore the relationship between different PA patterns and levels and COPD, the levels of each PA pattern were classified into three categories based on adherence to PA guidelines: no PA (0 MET-min/week), insufficient PA (<600 MET-min/week), and sufficient PA (≥600 MET-min/week) (12, 15).

### 2.3 Covariates

Other covariates in this study included gender, age at the time of the survey, race/ethnicity, family income-to-poverty ratio (PIR), education level, smoking status, drinking status and BMI levels, which were obtained through demographic questionnaires and physical measurements. Race/ethnicity was grouped into Mexican American, non-Hispanic white, non-Hispanic black, other Hispanic, and others. Educational level was classified as less than high school, high school or equivalent, and college or above. Family PIR was calculated by dividing family income according to the poverty guidelines and further divided into 4 categories (0–1.3, −1.8, −3.0, and>3.0). The lifestyle factors include smoking status and drinking status. Smoking status was categorized based on self-reported cigarette consumption: participants who had smoked fewer than 100 cigarettes in their lifetime were classified as “never smokers,” those who had reported smoking ≥100 cigarettes and currently smoke either “daily” or “occasionally” were designated as “current smokers,” and participants who had reported smoking ≥100 cigarettes but had quit at the survey were identified as “former smokers” (22). Participants who had never consumed more than 12 alcoholic beverages throughout their lifetime were categorized as non-drinkers, while those who had consumed exactly 12 drinks but abstained from alcohol in the past year were classified as former drinkers (23). Current drinkers refer to participants who had consumed at least 12 drinks in their lifetime and had at least one drink within the previous year. The BMI at the survey was divided into four categories (<18.5 kg/m2, 18.5–25 kg/m2, 25–30 kg/m2, and ≥30 kg/m2).

### 2.4 Statistical analysis

Considering the complex sampling design of NHANES, this study incorporated sampling weights, stratification, and primary sampling units (PSU) in all analyses to ensure nationally representative estimates. For continuous variables, population characteristic data were represented by mean and standard deviation (SD); while categorical variables were represented by frequency (n) and proportion (%).

The multivariable logistic regression model was used to explore the independent relationship between PA level or pattern and COPD. Total PA levels were referenced to insufficiently active populations, and each PA pattern was referenced to populations lacking the corresponding PA pattern. Model 1 did not adjust for potential confounding factors. In Model 2, we adjusted for age and gender as covariates. And in Model 3, we further adjusted for race/ethnicity, education level, PIR level, smoking status, and drinking status. Moreover, we combined PA of different intensities but the same type of activity to further analyze the relationship between them and COPD, in order to test the robustness of our findings.

Predefined subgroup analyses and potential effect modifications were conducted by survey age (< 60 and≥60 years), gender (Male and Female), smoking status (Non-smoker and Smoker) and BMI level (< 25 kg/m2 and ≥25 kg/m2). No adjustment for multiplicity in the subgroup analysis was done because of the exploratory design of this study part. We also used restricted cubic spline (RCS) to evaluate the nonlinear relationship between total PA level and COPD, with three knots at the 5th, 50th, and 95th.

All statistical analyses were conducted in 2024 using SAS 9.4 (SAS Institute Inc., Cary, NC, USA). The forest plot was made by the “forestplot” package in R 4.0.2 (R Foundation for Statistical Computing, Vienna, Austria). Statistical significance was defined as *p* < 0.05 using two-sided tests.

## 3 Results

### 3.1 Baseline characteristics and PA patterns

Table 1 presented the characteristics and distribution of PA patterns among the 34,392 participants included in this study analysis after weighted adjustment. Among them, 92.41% of participants were not diagnosed with COPD, while 7.59% had a diagnosis of COPD. The average age of the baseline sample was 47.7 years, with 51.37% being female and 48.63% being male. The study sample was 66.05% non-Hispanic white, 11.35% non-Hispanic black, 8.53% Mexican American, and 5.90% other Hispanic. In this sample, the number of participants engaged in vigorous work activity, moderate work activity, TPA (walking and cycling), vigorous recreational activity, and moderate recreational activity were 6,535, 12,171, 8,628, 7,392, and 13,469, respectively.

**Table 1:** Characteristics of study participants in NHANES 2007–2018 according to physical activity patterns^a^.

Characteristics	Overall	COPD	
		No	Yes
Participants	34,392	31,685 (92.41)	2,707 (7.59)
Age (mean ± SD^b^, years)	47.70 ± 0.23	46.99 ± 0.23	56.32 ± 0.41
Gender			
Male	16,858	15,691 (93.08)	1,167 (6.92)
Female	17,534	15,994 (91.22)	1,540 (8.78)
Race/ethnicity			
Mexican American	5,133	4,949 (96.42)	184 (3.58)
Other Hispanic	3,627	3,400 (93.74)	227 (6.26)
Non-Hispanic White	13,943	12,373 (88.74)	1,570 (11.26)
Non-Hispanic Black	7,382	6,859 (92.92)	523 (7.08)
Other	4,307	4,104 (95.29)	203 (4.71)
Education			
Less than high school	8,610	7,804 (90.64)	806 (9.36)
High school or equivalent	7,843	7,147 (91.13)	696 (8.87)
College or above	17,889	16,687 (93.28)	1,202 (6.72)
Family income-poverty ratio level			
0 ~ 1.3	9,863	8,798 (89.20)	1,065 (10.80)
~1.85	4,270	3,858 (90.35)	412 (9.65)
~3	5,533	5,112 (92.39)	421 (7.61)
>3	10,939	10,403 (95.10)	536 (4.90)
Smoking status			
Never	19,177	18,401 (95.95)	776 (4.05)
Former	8,191	7,230 (88.27)	961 (11.73)
Current	6,998	6,029 (86.15)	969 (13.85)
Drinking status			
Never	3,741	3,535 (94.49)	206 (5.51)
Former	1,694	1,523 (89.91)	171 (10.09)
Current	16,661	15,534 (93.24)	1,127 (6.76)
BMI status (kg/m^2^)			
<18.5	531	459 (86.44)	72 (13.56)
18.5 ≤ BMI<25	8,842	8,296 (93.82)	546 (6.18)
25 ≤ BMI<30	10,694	9,983 (93.35)	711 (6.65)
≥30	12,498	11,283 (90.28)	1,215 (9.72)
Physical activity patterns			
Vigorous work activity	6,535	6,039	496
Moderate work activity	12,171	11,163	1,008
Walk/bicycle for transportation (TPA)	8,628	8,145	483
Vigorous recreational activity	7,392	7,130	262
Moderate recreational activity	13,469	12,736	733

^a^All estimates accounted for complex survey designs. Data are expressed as No. (%) unless otherwise indicated.

^b^SD, Standard Deviation.

### 3.2 The association of total PA levels and COPD

Table 2 uses the insufficiently active population as the reference to analyze the relationship between total PA levels and COPD. In Model 3, adjusted for all covariates, being sufficiently active (OR: 0.86, 95% CI = 0.75–0.98, *p* = 0.025) and HEPA active (OR: 0.84, 95% CI = 0.73–0.96, *p* = 0.010) were associated with a lower prevalence of COPD.

**Table 2:** Odd ratios (OR) and 95% confidence intervals (CIs) of COPD with total physical activity level in the NHANES 2007–2018^a^.

Total physical activity level	No. of COPD/No. of participants	Model 1^b^		Model 2^c^		Model 3^d^	
		OR (95% CI)	*p*	OR (95% CI)	*p*	OR (95% CI)	*p*
Insufficiently active(ref)^e^	1755/18425	1.00		1.00		1.00	
Sufficiently active^f^	481/7829	0.63 (0.55, 0.72)	<0.001	0.74 (0.65, 0.84)	<0.001	0.86 (0.75, 0.98)	0.025
HEPA active^g^	471/8138	0.56 (0.50, 0.64)	<0.001	0.82 (0.72, 0.94)	0.004	0.84 (0.73, 0.96)	0.010

^a^All estimates accounted for complex survey designs.

^b^Model 1 was unadjusted.

^c^Model 2 was adjusted for age and gender.

^d^Model 3 was additionally adjusted for race/ethnicity, education level, family income-poverty ratio level, smoking status, and drinking status.

^e^Those individuals who not met criteria for note f or g were considered “Insufficiently active”.

^f^“Sufficiently active” was any one of the following 3 criteria: (1) 3 or more days of vigorous activity of at least 20 min per day OR (2) 5 or more days of moderate-intensity activity or walking of at least 30 min per day OR (3) 5 or more days of any combination of walking, moderate-intensity or vigorous intensity activities achieving a minimum of at least 600 MET-min/week.

^g^“HEPA (Health Enhancing Physical Activity) active” was one of the following 2 criteria: (1) vigorous-intensity activity on at least 3 days achieving a minimum of at least 1,500 MET-minutes/week OR (2) 7 or more days of any combination of walking, moderate-intensity or vigorous intensity activities achieving a minimum of at least 3,000 MET-minutes/week.

### 3.3 The dose-response relationship between total PA level and COPD

**Figure 2:**
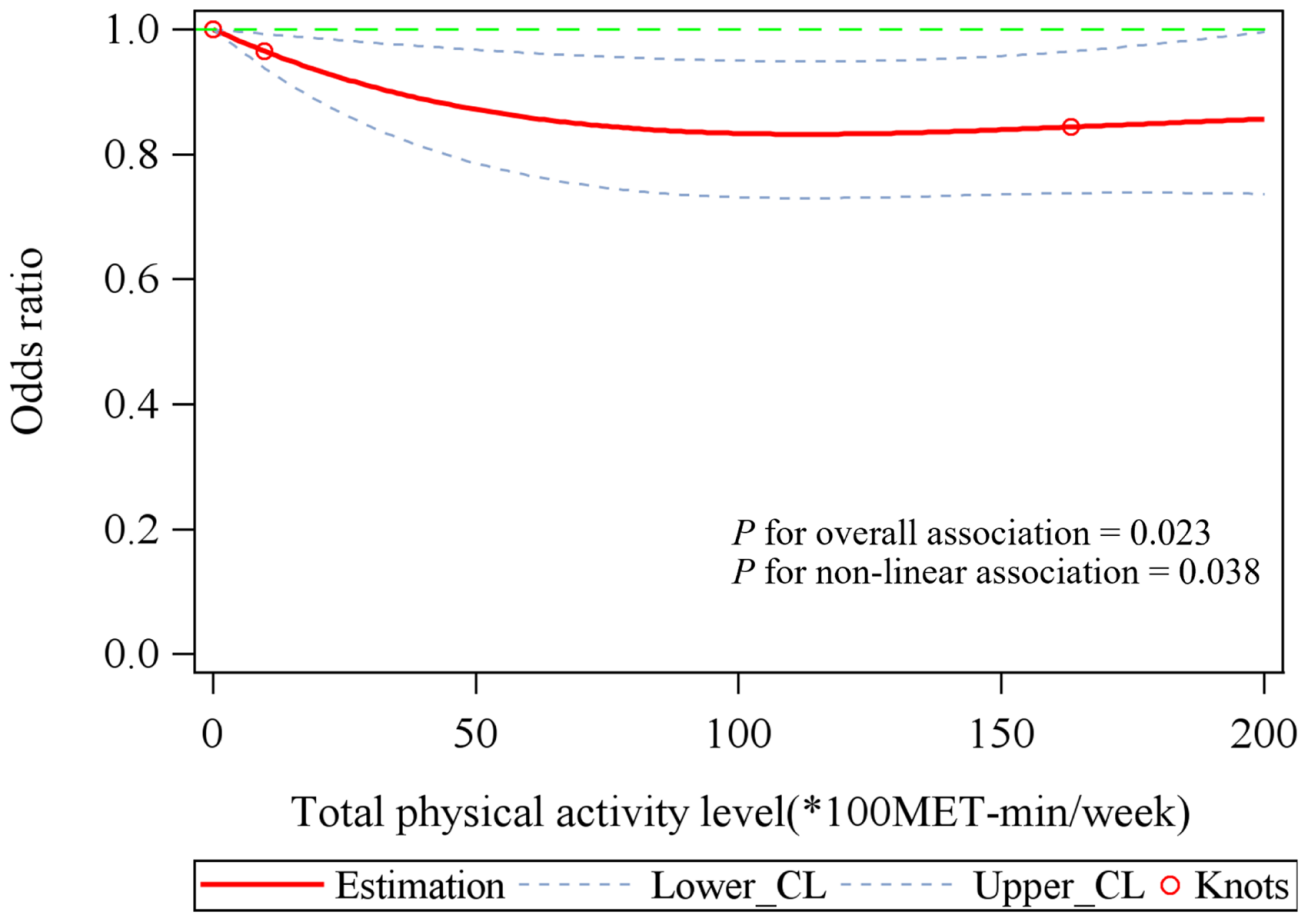
The association between total physical activity level and COPD. Associations were examined by multivariable logistic regression models based on restricted cubic splines. Red solid line represents estimates of odds ratios (reference value for total physical activity level: 0*100MET-min/week). Blue dashed line represents 95% confidence intervals. Red circle represents knots located at the 5th, 50th and 95th percentiles of the distribution of total physical activity level. Risk estimates were adjusted for age, gender, race/ethnicity, education level, family income-poverty ratio level, smoking status, and drinking status. *p* values for overall association was 0.023 and *p* values for non-linear association was 0.038.

To investigate the relationship between total PA levels and COPD, we constructed an RCS model based on Model 3 from Table 2. We found a significant non-linear relationship between total PA levels and COPD, with *p* values for overall association at 0.023 and *p* values for non-linear association at 0.038. Additionally, the RCS curve plateaued when total PA levels exceeded 5,000 MET-min/week (Figure 2).

### 3.4 The association of different PA patterns and levels and COPD

Table 3 and Supplementary Table S1 present the relationships between different PA patterns, levels, and COPD, using populations lacking corresponding PA patterns as references. Table 3 categorizes PA into 5 types, each divided into 3 levels: 0 MET-min/week (no PA), <600 MET-min/week (insufficient PA), and ≥600 MET-min/week (sufficient PA). In Model 3, adjusted for all covariates, sufficient vigorous work activities showed no significant association with COPD, while low (OR: 1.35, 95% CI = 1.12–1.62, *p* = 0.002) and sufficient moderate work activities (OR: 1.19, 95% CI = 1.05–1.35, *p* = 0.006) were associated with higher COPD prevalence. Conversely, sufficient TPA (OR: 0.72, 95% CI = 0.59–0.89, *p* = 0.003), sufficient vigorous recreational activities (OR: 0.68, 95% CI = 0.55–0.85, *p* < 0.001), low (OR: 0.77, 95% CI = 0.66–0.90, *p* = 0.001), and sufficient moderate recreational activities (OR: 0.71, 95% CI = 0.61–0.84, *p* < 0.001) were significantly associated with lower COPD prevalence.

**Table 3:** Odd ratios (OR) and 95% confidence intervals (CIs) of COPD with different physical activity patterns and levels in the NHANES 2007–2018^a^.

Physical activity patterns	No. of COPD/No. of participants	Model 1^b^		Model 2^c^		Model 3^d^	
		OR (95% CI)	*p*	OR (95% CI)	*p*	OR (95% CI)	*p*
Vigorous work activity							
0 MET-min/week(ref)	2205/27786	1.00		1.00		1.00	
<600 MET-min/week	88/799	1.08 (0.84, 1.40)	0.541	1.29 (0.99, 1.67)	0.056	1.22 (0.93, 1.60)	0.142
≥600 MET-min/week	408/5736	0.92 (0.80, 1.05)	0.201	1.28 (1.11, 1.49)	0.001	1.10 (0.94, 1.28)	0.237
Moderate work activity							
0 MET-min/week(ref)	1686/22118	1.00		1.00		1.00	
<600 MET-min/week	263/2659	1.25 (1.05, 1.49)	0.015	1.29 (1.08, 1.53)	0.005	1.35 (1.12, 1.62)	0.002
≥600 MET-min/week	745/9512	1.04 (0.92, 1.17)	0.572	1.28 (1.13, 1.46)	<0.001	1.19 (1.05, 1.35)	0.006
Walk/bicycle for transportation (TPA)							
0 MET-min/week(ref)	2216/25715	1.00		1.00		1.00	
<600 MET-min/week	226/3846	0.70 (0.57, 0.86)	<0.001	0.83 (0.68, 1.02)	0.077	0.83 (0.68, 1.03)	0.088
≥600 MET-min/week	257/4782	0.62 (0.51, 0.76)	<0.001	0.76 (0.62, 0.94)	0.013	0.72 (0.59, 0.89)	0.003
Vigorous recreational activity							
0 MET-min/week(ref)	2441/26979	1.00		1.00		1.00	
<600 MET-min/week	54/1224	0.46 (0.30, 0.70)	<0.001	0.67 (0.43, 1.03)	0.069	0.91 (0.57, 1.44)	0.670
≥600 MET-min/week	208/6168	0.33 (0.27, 0.41)	<0.001	0.48 (0.38, 0.60)	<0.001	0.68 (0.55, 0.85)	<0.001
Moderate recreational activity							
0 MET-min/week(ref)	1968/20885	1.00		1.00		1.00	
<600 MET-min/week	358/6808	0.55 (0.47, 0.64)	<0.001	0.61 (0.52, 0.72)	<0.001	0.77 (0.66, 0.90)	0.001
≥600 MET-min/week	375/6661	0.56 (0.48, 0.66)	<0.001	0.60 (0.51, 0.70)	<0.001	0.71 (0.61, 0.84)	<0.001

^a^All estimates accounted for complex survey designs.

^b^Model 1 was unadjusted.

^c^Model 2 was adjusted for age and gender.

^d^Model 3 was additionally adjusted for race/ethnicity, education level, family income-poverty ratio level, smoking status, and drinking status.

Supplementary Table S1 consolidates the 5 PA types into 3 categories (OPA, TPA, RPA), further validating the robustness and sensitivity of the findings. In Model 3, low (OR: 1.31, 95% CI = 1.09–1.57, *p* = 0.004) and sufficient OPA (OR: 1.20, 95% CI = 1.06–1.35, *p* = 0.003) were associated with higher COPD prevalence, while low RPA (OR: 0.81, 95% CI = 0.68–0.97, *p* = 0.021), sufficient RPA (OR: 0.67, 95% CI = 0.58–0.78, *p* < 0.001), and sufficient TPA (OR: 0.72, 95% CI = 0.59–0.89, *p* = 0.003) were associated with lower COPD prevalence.

### 3.5 Stratified analyses according to demographics characteristics

**Figure 3:**
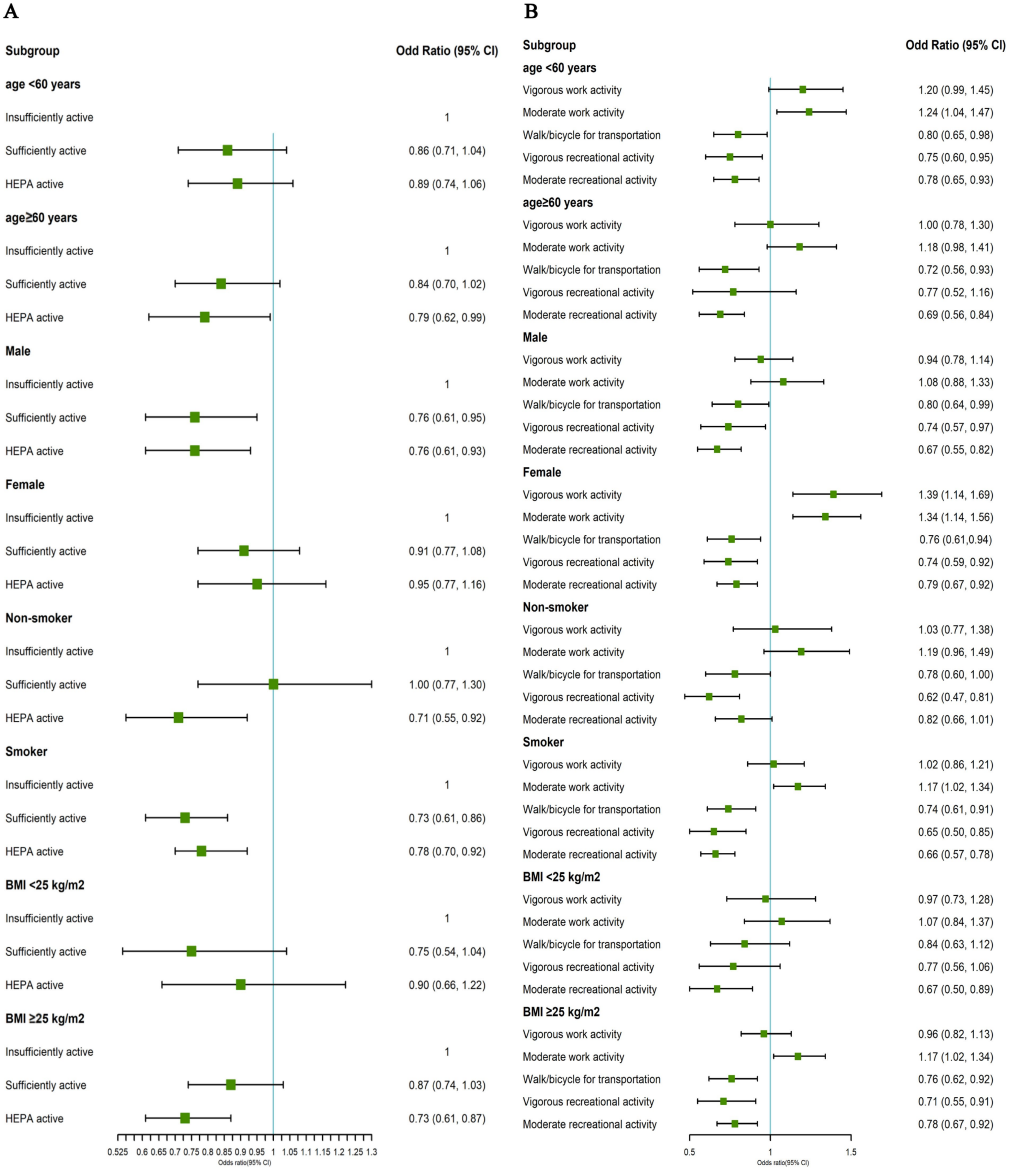
**(A)** Associations between total physical activity levels and COPD. **(B)** Associations between physical activity patterns and COPD. All estimates accounted for complex survey design of NHANES and stratified by age, gender, smoking status and BMI status in NHANES 2007–2018. Risk estimates were adjusted for age (not adjusted in subgroup analysis by age), gender (not adjusted in subgroup analysis by gender), race/ethnicity, education level, family income-poverty ratio level, smoking status (not adjusted in subgroup analysis by smoking status), drinking status and BMI (not adjusted in subgroup analysis by BMI status).

In the stratified analysis of three total PA levels, we found a significant interaction between being sufficiently active and smoking status (P for interaction = 0.013). Being sufficiently active showed a significant negative association with COPD prevalence among smokers (OR: 0.73, 95% CI = 0.61–0.86) but showed no significant association among non-smokers (OR: 1.00, 95% CI = 0.77–1.30). Additionally, being HEPA active showed an interaction with gender (P for interaction = 0.023). Compared to females (OR: 0.95, 95% CI = 0.77–1.16), males maintaining HEPA active levels showed a stronger negative association with COPD prevalence (OR: 0.76, 95% CI = 0.61–0.93) (Figure 3A).

In the stratified analysis of five PA types, we observed significant interactions between vigorous work activities (P for interaction<0.001), moderate work activities (P for interaction = 0.040), and gender. Interestingly, the association between OPA and COPD prevalence was more pronounced in females (vigorous work activities: OR: 1.39, 95% CI = 1.14–1.69; moderate work activities: OR: 1.34, 95% CI = 1.14–1.56) but not significant in males (vigorous work activities: OR: 0.94, 95% CI = 0.78–1.14; moderate work activities: OR: 1.08, 95% CI = 0.88–1.33). Furthermore, moderate recreational activities showed a significant interaction with smoking status (P for interaction = 0.008). Compared to non-smokers (OR: 0.82, 95% CI = 0.66–1.01), smokers engaging in moderate recreational activities had a stronger association with lower COPD prevalence (OR: 0.66, 95% CI = 0.57–0.78) (Figure 3B).

## 4 Discussion

In this large, nationally representative retrospective study of U.S. adults, we examined the relationship between PA levels, patterns, and COPD. We observed that the RCS curve began to plateau when total PA levels exceeded 5,000 MET-min/week. However, achieving 5,000 MET-min/week requires approximately 10.42 h (625 min) of high-intensity exercise per week, equivalent to about 1.5 h of daily high-intensity running (8 km/h, 5 mph) sustained over 7 days. This level of PA is not only challenging for most individuals to maintain but may also pose potential health risks. Even athletes, who may approach this level during peak training periods, would find it difficult to sustain over the long term. We also found a nonlinear relationship between total PA levels and COPD within the range of 0–5,000 MET-min/week, characterized by a negative correlation. Additionally, PA patterns were closely associated with COPD. After adjusting for other covariates, other PA patterns continued to show statistical associated with COPD, except for vigorous work activities. We observed that the proportion of smokers was significantly higher among those engaged in vigorous work activities compared to those who were not, which may have influenced the results. Furthermore, TPA and vigorous recreational activities were associated with a lower prevalence of COPD only when performed at sufficient levels. However, our pooled data indicated that TPA and RPA were associated with a lower prevalence of COPD, while OPA was associated with a higher prevalence of COPD. In stratified analyses, we found that the correlation between maintaining high levels of total PA (HEPA active) and a lower COPD prevalence was more pronounced in males than in females. Notably, the association between OPA and COPD was more evident in females compared to males. Moreover, among smokers, maintaining sufficiently active PA levels and engaging in moderate recreational activities showed a stronger association with a reduced COPD prevalence than in non-smokers.

The health benefits of PA have been extensively studied and well-documented. A substantial body of literature indicates that being physically active significantly prevents and improves various chronic diseases, such as cardiovascular disease, diabetes, obesity, and certain cancers (24). PA is also important for COPD. Research shows that PA can reduce the risk of COPD and improve patients’ quality of life through mechanisms such as enhancing lung function, improving cardiorespiratory fitness, and reducing systemic inflammation and oxidative stress (25). However, our study revealed a nonlinear relationship between PA and COPD weakened significantly after exceeding 5,000 MET-min/week. This finding suggests that we should not blindly pursue high-intensity PA but instead focus more on the appropriate intensity and sustainability of PA. High-intensity exercise is not only difficult for most people to achieve but may also be detrimental to health. O’Donovan et al. (26) demonstrated that excessive exercise may lead to myocardial injury and chronic fatigue, thereby increasing the risk of cardiovascular events. For middle-aged and older adults, high-intensity PA also increases the risk of exercise-related injuries, such as muscle strains and joint damage. In contrast, moderate-intensity PA is more suitable for the majority of the population, easier to maintain, and more beneficial to overall health. Lee et al. (6) through a large-scale systematic review and meta-analysis, evaluated the relationship between PA and all-cause mortality, finding that moderate-intensity PA (e.g., brisk walking, leisure cycling, or gardening) was associated with a significant reduction in all-cause mortality, with health benefits comparable to those of high-intensity PA. Therefore, we still recommend moderate-intensity PA as the preferred strategy for health promotion and maintaining a “Sufficiently Active” or “HEPA Active” PA level, rather than pursuing excessively high PA levels.

In recent years, numerous studies from different countries have shown significant differences in the health impacts of various types of PA. For example, individuals lacking RPA face higher risks of prediabetes, type 2 diabetes, metabolic syndrome, and depression (15, 27–29). In contrast to RPA, OPA is a risk factor for many diseases, such as chronic kidney disease and cancer (30, 31). TPA has effects similar to RPA, not only providing the aerobic activity the body needs but also significantly reducing the risks of cardiovascular disease, type 2 diabetes, and obesity (32). Our study found that the association between PA and COPD is not only closely related to total PA levels but also to PA patterns and their respective intensities. Consistent with previous findings on other diseases, RPA and TPA were negatively associated with COPD prevalence, while OPA was positively associated with COPD prevalence. This difference may stem from the unique nature of OPA: OPA is often performed passively to sustain livelihoods and is frequently accompanied by specific occupational exposures (e.g., dust, chemicals, harmful gases, or noise), which may adversely affect health (33). Additionally, the repetitive and sustained nature of work activities may lead to chronic fatigue and respiratory burden, further exacerbating the impact on COPD. However, unlike the positive correlation between moderate work activities and COPD prevalence, we did not find a significant association between vigorous work activities and COPD. This result may be due to the higher proportion of smokers among those engaged in vigorous work activities, which could mask the true effect of vigorous work activities on COPD and introduce confounding effects. In contrast, RPA is an activity individuals engage in voluntarily, driven by intrinsic motivation, and is associated with higher levels of enjoyment and psychological satisfaction. Our study found that sufficient RPA (≥600 MET-min/week) was significantly associated with a lower COPD prevalence. Beyond environmental factors related to PA, self-determined motivation may also explain this difference (34). Regular RPA may also induce changes in gene transcription within the body, affecting the production of neurotransmitters such as dopamine, thereby further activating intrinsic vitality and self-regulatory capacity (35, 36). Moreover, sufficient TPA (≥600 MET-min/week), as a sustainable moderate-intensity PA, was also significantly associated with a lower COPD prevalence. TPA not only provides health benefits similar to RPA but also directly reduces air pollution, indirectly influencing COPD. Therefore, the association between PA and COPD is multidimensional, and we advocate for increasing RPA and TPA outside of work to balance COPD health risks.

It is noteworthy that PA shows significant associations with lung function in smokers. Menegali et al. (37) found that physical training could partially improve lung histology and oxidative stress parameters in animals chronically exposed to cigarette smoke, suggesting that regular PA helps mitigate the harmful effects of smoking. Additionally, the impact of PA on lung function varies with different PA levels. Luzak et al. (38) demonstrated that physically active individuals exhibited higher lung capacity compared to inactive individuals, and this association was more pronounced among smokers. A prospective study further indicated that moderate to high levels of regular PA could ameliorate smoking-related declines in lung function and reduce the risk of COPD (25). Another study found that vigorous leisure-time PA was associated with higher FEV1 and FVC, and these associations were more significant in smokers than in non-smokers (39). These findings align with our study results, which show a significant negative correlation between recreational activity and COPD prevalence among smokers. This relationship may stem from various physiological mechanisms induced by PA, such as upregulating antioxidant defense systems and releasing anti-inflammatory factors, thereby partially offsetting the negative impact of smoking on lung function (25, 40, 41).

Our stratified analysis also revealed that, compared to males, females engaged in OPA were associated with a higher prevalence of COPD. This difference may be related to conflicts in social roles: in addition to occupational physical labor, females often bear more household chores and childcare responsibilities. This dual burden may lead to prolonged exposure to high-intensity PA and psychological stress, thereby negatively impacting health and increasing the risk of COPD (42). Therefore, the conflict of social roles and the dual impact of occupational exposures may be significant reasons for the stronger association between OPA and COPD prevalence in females.

Our study had several strengths. First, the inclusion of a large, nationally representative sample enables us to evaluate the relationship between different PA patterns and levels and COPD. Second, the NHANES participants were selected from a general, non-institutionalized population, making our results highly generalizable. Additionally, the detailed data collection in NHANES allowed us to adjust for several important confounding factors that could influence the outcomes, thereby enhancing the generalizability of our findings.

Our study also had several limitations. Firstly, PA was self-reported by participants, which may introduce recall bias in our study. However, questionnaires remain the mainstream method for existing surveys. Secondly, we only included participants aged 20 years or older and excluded those who were pregnant, so the findings may not be generalizable to other age groups or special populations. Thirdly, we used self-reported data for COPD status, which may have missed individuals who were undiagnosed. Fourthly, our study is cross-sectional, with PA data collected only once and no time frame defined for COPD diagnosis, making it impossible to account for changes over time or establish a causal relationship between PA and COPD risk. Lastly, we only examined the relationship between individual PA patterns, levels, and total PA levels with COPD. Due to the large number of participants and the complexity of PA pattern combinations, further validation of the relationship between various PA pattern combinations and COPD was not conducted. Future prospective studies based on PA monitoring are needed to confirm the impact of different PA patterns and levels on COPD.

## 5 Conclusion

In U.S. adults, there is a non-linear relationship between total PA levels and COPD. Additionally, different PA patterns and levels are differentially associated with COPD. In this study, TPA and RPA were associated with a lower prevalence of COPD, while OPA was associated with a higher prevalence of COPD. However, it is important to note that COPD patients may reduce their activity levels due to disease symptoms, which could influence the interpretation of the study results. Therefore, increasing the relative proportion of TPA and RPA within total PA may be a feasible strategy associated with reducing COPD risk, but further research is needed to validate the causal relationship.

## Data Availability

The datasets presented in this study can be found in online repositories. The names of the repository/repositories and accession number(s) can be found at: https://www.cdc.gov/nchs/nhanes/index.htm.
