# Supplementary material for: Relationship between physical activity and chronic obstructive pulmonary disease: a cross-sectional study
Source: Front Public Health. 2025 May 16;13:1583265. doi: 10.3389/fpubh.2025.1583265 (PMC12122498; doi:10.3389/fpubh.2025.1583265)
Supplement: Supplementary file 1 [file Table_1.docx]

Supplementary Material

# Supplementary Table 1. Odd ratios (OR) and 95% confidence intervals (CIs) of COPD with combined physical activity patterns in the NHANES 2007-2018. ^a^

| **Physical activity patterns** | **No. of COPD / No. of participants** | **Model 1 ^b^** | | **Model 2 ^c^** | | **Model 3 ^d^** | |
| --- | --- | --- | --- | --- | --- | --- | --- |
|  |  | **OR (95% CI)** | ***P*** | **OR (95% CI)** | ***P*** | **OR (95% CI)** | ***P*** |
| **Moderate-to-vigorous work activity (OPA)** | | | | | | | |
| 0 MET-min/week(ref) | 1597/20720 | 1 |  | 1 |  | 1 |  |
| ＜600 MET-min/week | 248/2458 | 1.21 (1.02, 1.44) | 0.033 | 1.26 (1.05, 1.50) | 0.012 | 1.31 (1.09, 1.57) | 0.004 |
| ≥600 MET-min/week | 857/11165 | 1.03 (0.92, 1.15) | 0.655 | 1.32 (1.17, 1.48) | <0.001 | 1.20 (1.06, 1.35) | 0.003 |
| **Walk/bicycle for transportation (TPA)** | | | | | | | |
| 0 MET-min/week(ref) | 2216/25715 | 1 |  | 1 |  | 1 |  |
| ＜600 MET-min/week | 226/3846 | 0.70 (0.57, 0.86) | <0.001 | 0.83 (0.68, 1.02) | 0.077 | 0.83 (0.68, 1.03) | 0.088 |
| ≥600 MET-min/week | 257/4782 | 0.62 (0.51, 0.76) | <0.001 | 0.76 (0.62, 0.94) | 0.013 | 0.72 (0.59, 0.89) | 0.003 |
| **Moderate-to-vigorous recreational activity (RPA)** | | | | | | | |
| 0 MET-min/week(ref) | 1873/18417 | 1 |  | 1 |  | 1 |  |
| ＜600 MET-min/week | 322/5285 | 0.60 (0.51, 0.71) | <0.001 | 0.67 (0.56, 0.79) | <0.001 | 0.81 (0.68, 0.97) | 0.021 |
| ≥600 MET-min/week | 510/10684 | 0.41 (0.35, 0.49) | <0.001 | 0.51 (0.44, 0.60) | <0.001 | 0.67 (0.58, 0.78) | <0.001 |

^a^ All estimates accounted for complex survey designs.

^b^ Model 1 was unadjusted.

^c^ Model 2 was adjusted for age and gender.

^d^ Model 3 was additionally adjusted for race/ethnicity, education level, family income-poverty ratio level, smoking status, and drinking status.
